# At the heart of peer review: why artificial intelligence concordance misses the human pulse

**DOI:** 10.1093/ehjimp/qyag127

**Published:** 2026-07-20

**Authors:** Zekai Yu

**Affiliations:** School of Computer Science and Technology, Hangzhou Dianzi University, No. 1158, No. 2 Road, Xiasha Higher Education Zone, Hangzhou, Zhejiang 310018, PR China

The rapid introduction of artificial intelligence (AI) into the peer review process has ignited a necessary debate regarding the evaluation of manuscript value.^1^ Earlier work has already established the potential of AI-assisted peer review,^2^ but empirical evidence in clinical journals is rapidly evolving. Zancanaro *et al.*^3^ recently demonstrated that large language models (LLMs) can achieve a 67.5% concordance rate with final editorial decisions, proving non-inferior to the human consensus of 71.9%. Furthermore, their blinded comparative study highlighted that AI-generated reviews scored significantly higher than their human counterparts in structured quality domains such as focus, balance, precision, and conclusiveness, and were produced in mere minutes compared to a median human turnaround time of 17 days. This aligns with large-scale empirical analyses showing that LLMs can provide highly useful and structured feedback on research papers.^4^

While these operational efficiencies are undeniably attractive for overburdened editorial workflows, we must critically examine the reference standard used to assess AI performance. As Matsubara^1^ astutely points out, the actual acceptance or rejection of a manuscript does not necessarily equate to its intrinsic scientific value. Editorial decisions are inherently influenced by a myriad of pragmatic factors, including journal policy, journal-manuscript matching, and available space. If we validate AI solely by its ability to replicate existing editorial outcomes, we risk training these systems to mimic human systemic biases rather than objectively recognizing true clinical usefulness.

Furthermore, focusing heavily on structured evaluation domains and turnaround times overlooks the fundamental, qualitative essence of peer review: scientific mentorship. Peer review is not merely a triage mechanism designed to filter submissions for editors; it is an iterative dialogue between colleagues meant to nurture and elevate research. An AI may provide highly balanced and precise methodological critiques,^3^ but it lacks the contextual understanding, empathy, and visionary capacity to mentor a researcher through a complex conceptual revision.

Matsubara argues that AI might eventually identify value differently, and perhaps even better, than human reviewers. We propose that until AI can fully comprehend the evolving definition of ‘value’, we must adopt a formalized hybrid workflow. This concept of comparing human and AI expertise to move towards a complementary approach is increasingly supported in recent literature.^5^ As detailed in *Table 1*, we suggest a structured division of labour where AI acts as the preliminary methodological validator, freeing human reviewers to focus on conceptual novelty, ethical implications, and constructive mentorship.

**Table 1:** Proposed division of peer review labour in a hybrid AI-human workflow

Evaluation domain	AI primary role	Human primary role
Methodological precision	High (data integrity, statistical checks)	Moderate (contextual validity)
Tone and politeness	Consistently high	Variable
Novelty assessment	Low (constrained by training data bounds)	High (awareness of paradigm shifts)
Constructive mentorship	Low (formulaic suggestions)	High (developmental dialogue)

If, as Matsubara^1^ advocates, publishing 10 papers requires a reciprocal commitment of at least 20 reviews, integrating AI as a technical pre-reviewer could make this give-and-take process far more sustainable. AI-generated reviews should serve as a specialized lens, complementing rather than replacing individualized human assessment. The true value of AI in scientific publishing will ultimately be measured not by how perfectly it agrees with editors,^3^ but by how effectively it empowers human reviewers to elevate scientific discourse.

## Data Availability

Data sharing is not applicable to this article as no new data were created or analysed in this study.

## Lead author biography

Zekai Yu is an undergraduate researcher at the School of Computer Science and Technology, Hangzhou Dianzi University, specializing in biomedical AI and causal machine learning. His primary research focuses on unravelling complex treatment effects through causal inference models and developing reliable AI architectures for clinical decision support systems. As an active peer reviewer for over 40 medical and informatics journals, his work critically examines algorithmic bias, interpretability, and the practical challenges of integrating AI methodologies into both healthcare delivery and scientific peer review.
